# Violent Discipline in North Kivu, Democratic Republic of Congo: The Role of Child Gender and Disability Status in Cross-sectional Analysis

**DOI:** 10.1007/s10995-023-03598-4

**Published:** 2023-02-28

**Authors:** Alexandra H. Blackwell, Jean de Dieu Hategekimana, Daddy Bauma, Danielle Roth, Pauline Thivillier, Meghan O’Connor, Kathryn Falb

**Affiliations:** 1 International Rescue Committee, 122 E 42nd St, New York, NY 10168 USA; 2 International Rescue Committee Democratic Republic of Congo, 8 Avenue des Citronniers, Q. Croiz-Rouge, Kinshasa/Gombe, RD Congo, BP 8119 South Africa; 3 Department of Social Policy and Intervention, University of Oxford, Barnett House, 32-37 Wellington Square, Oxford, OX1 2ER United Kingdom

**Keywords:** Child abuse, Violent discipline, Disability, Gender attitudes, Parenting

## Abstract

**Introduction:**

Violence is used to punish or educate children across the world, with detrimental effects on their physical, emotional, and social health that persist into their adulthood. This study aimed to understand the use of violent discipline by caregivers in conflict-affected communities and how it varied by the child’s gender and disability level.

**Methods:**

Using cross-sectional data collected from 394 respondents (196 men and 198 women) in North Kivu, Democratic Republic of Congo, logistic generalized estimating equations stratified by gender assessed the association between past-three-month perpetration of violent discipline, caregiver demographics, conflict experiences, and disability attitudes, as well as child demographics of age, gender, disability level, and the interaction of gender and disability.

**Results:**

Compared to women with boy children with no disability, odds of perpetration of violent discipline were higher among those with girl children with no disability (aOR: 2.24; 95%CI: 1.11–4.51) and boy children with moderate or severe disability (aOR: 2.91; 95%CI: 1.14–7.33), and the interaction of girl children with a moderate or severe disability showed a 7.80 increase in odds of perpetration; however, association with women’s discriminatory disability attitudes was not significant. In contrast, the interaction of child gender and disability level were not significantly associated with perpetration of violent discipline for men, but disability attitudes were significantly associated (aOR: 1.07; 95%CI: 1.00-1.15).

**Discussion:**

Results suggest that levels of violence in conflict-affected households in North Kivu, DRC are high, with women reporting higher levels of violent discipline overall, and amplified use of violence against girl children with disabilities. More research and programs with an intersectional lens are needed in conflict settings to better understand and address the use of violent discipline and underlying discriminatory norms around gender and disability.

## Significance

Violence against children in the home varies by gender and disability level. Evidence from predominantly Global North contexts demonstrates that children with disabilities are more likely to experience violence, with heightened risk for girls. Among conflict-affected communities in DRC, women had higher odds of using violence against girls and children with disabilities, and even higher for girl children with disabilities. Violence perpetration was also associated with the caregivers’ conflict experiences. This suggests that to strengthen violence prevention in conflict settings, research and programs must be more accessible to and inclusive of people with disabilities and address inequitable gender norms.

## Introduction

Globally, an estimated four out of five children between the ages of 2–14 experience violent discipline, and over one in four caregivers say that physical punishment is necessary to properly raise or educate children (UNICEF, 2017). This violence has detrimental effects on children, impeding their physical, emotional, and social development (Chait et al. 2016; Guedes et al., 2016), and triggering economic and social consequences that last far beyond childhood (Krug et al., 2002; Pinheiro, 2006).

Children’s experiences of violence are influenced by their identities, which can evolve over time. Violence against children in the home, including violent discipline, varies by age and gender (Maternowska et al., 2016). Disability level can also heighten the risk of physical, sexual, and psychological violence for a child. A meta-analysis of 17 studies found that children with disabilities are almost four times more likely to be survivors of physical, emotional, and/or sexual abuse than are children without disabilities (Jones et al., 2012), and risk is even higher for those children with psychosocial or intellectual disabilities (Jones et al., 2012; Maclean et al., 2017). Girls with disabilities have a magnified risk of violence and maltreatment (Stark et al., 2020; van der Heijden & Dunkle, 2017). A qualitative study in South Africa found that women and girls with disabilities had experienced physical, psychological, and sexual gender-based violence (GBV) associated with disability-related stigma from a very young age, often at the hands of their caregivers and other family members in the home (van der Heijden et al., 2019).

While there remains a dearth of robust evidence on the maltreatment of girls and boys with disabilities outside of Global North countries (Jones et al., 2012; Leeb et al., 2012), literature from humanitarian settings demonstrates that conflict and displacement further exacerbate risk factors of violence against children (Guedes et al., 2016; Maternowska et al., 2016; Rubenstein et al., 2020; Rubenstein & Stark, 2017). Direct conflict experiences, coupled with changes in economic status, decline in mental health and coping strategies, and disruption to critical social support and infrastructure leave women, girls, and boys at highest risk of violence within their homes (Catani 2009; Parcesepe et al., 2016; Stark et al., 2017; Rubenstein et al., 2020). Similar to non-conflict settings, this violence may be magnified for children with disabilities, those of younger ages, and for girls with or without disabilities (Murphy et al., 2019; Stark et al., 2020). Further, given the breakdown in services and support structures and reduced access to trusted adults such as teachers, many cases of violence in humanitarian settings go unreported, particularly when perpetrated against children by caregivers or relatives (Pinheiro, 2006; Stark et al., 2020). Overall, understanding of experiences of abuse amongst children in humanitarian settings and how it may vary across different identities is limited.

To address this gap, this study utilizes baseline data from the trial of a family violence prevention program, Safe at Home (IRC, 2021), in North Kivu, DRC to increase understanding of an intersectional approach to assessing violent discipline across age, gender, and disability level. Now a complex humanitarian context, eastern DRC has suffered fluctuations in conflict since the region was colonized by Belgium, and recent conflicts have been compounded by the Ebola epidemic. While evidence on the prevalence of violent discipline within the home from eastern DRC is limited, one retrospective study found that violence experienced during childhood is pervasive, with over half of adult women and men experiencing physical, psychosocial or economic, or sexual violence at home as a child (women: 59.7%, 81.4%, and 29.7%; men: men: 58.9%, 79.3%, 35.4%) (Slegh, Barket, and Levtov 2014). The specific objectives of these analyses are to: (1) understand the frequency of violent discipline perpetration by men and women; (2) understand how risk of violent discipline may vary based on child age, gender, and disability; and (3) determine whether child gender and disability interact to increase risk of violence.

## Methods

### Study Design & Sample

The analyses in this paper use baseline quantitative data from a cluster randomized controlled trial of Safe at Home, a family-based intervention developed by the International Rescue Committee which aims to prevent and respond to co-occurring intimate partner violence (IPV) and child maltreatment within the home (Falb et al., 2022). Recruitment of programme participants occurred in November 2019. Community members interested in participating were introduced to the intervention by International Rescue Committee staff and assessed for eligibility. Inclusion criteria for the program were: being 18 years of age or older; living with an intimate partner who also agreed to register for the program; being part of a monogamous couple; having at least one child aged 6–12 years; and speaking Swahili, Kinyarwanda, or French. At enrollment, 406 individuals (203 couples) were registered in the program. All eligible program participants including both partners within the couple were invited to take part in the study, but both partners did not have to consent to partake in the survey. Baseline data were collected from 394 participants (196 men and 198 women) across four sites in North Kivu, DRC between November 2019 and January 2020. Of 406 individuals enrolled in the program and invited to participate in the study, only 12 people (7 men and 5 women) declined to participate in the baseline survey for a refusal rate of 2.96%.

### Data Collection

The survey was administered by gender-matched enumerators who read the questions aloud to the respondent and recorded their response in a tablet using the application SurveyCTO. Male and female enumerators were hired for the study and were trained on survey methodology, principles of confidentiality and privacy, informed consent, and concepts of gender-based violence, violence against children, and referral pathways. Separate survey tools were used for men and women, and all tools were translated and back translated into French from English and then translated into Swahili and Kinyarwanda. Translation into the local languages was further revised during the data collection training with enumerators to ensure clarity and consistency in meaning and interpretation of each question.

### Ethics

The research was conducted in accord with global ethical standards for research on violence (WHO, 2007). Interviews were conducted in a private space to protect participants’ confidentiality. Enumerators obtained the participants’ informed consent and made substantial efforts to ensure that participants understood their decision to participate would have no influence on their ability to receive services from the International Rescue Committee or other service providers. All respondents received information for follow-up services. The study protocol and tools were approved by the International Rescue Committee’s Institutional Review Board (WPE 1.00.014) and by the DRC Ministry of Public Health’s Ethics Committee (Comité National d’Ethique de la Santé) and have therefore been performed in accordance with the ethical standards laid down in the 1964 Declaration of Helsinki and its later amendments.

### Measures

The key measures used in the analyses are presented in Table 1. Violent discipline is defined for the study as caregiver perpetration of physical and/or psychological violent discipline and was measured using the Multiple Indicator Cluster Surveys (MICS) discipline module (UNICEF, 2013). Both women and men within the couple were asked to describe their own behavior regarding violent discipline in the past three months based on the index child (oldest child aged 6–12 years).

**Table 1:** Constructs assessed within the study

Construct	Measure	Construction/Scale	Example items	Source
Violent discipline perpetration (any violent discipline)	Binary summary variable indicating an affirmative response (yes) to any act of physical or psychological violent discipline against the index child (oldest child aged 612 years); it does NOT include non-violent discipline	8 binary items (yes/no) assessing past 3-month physical and psychological violent discipline	Please tell me if you have used this method with [name of child] in the past 3 months: • Hit (him/her) on the bottom or elsewhere on the body with something like a belt, hairbrush, stick or other hard object. • Called (him/her) dumb, lazy or another name like that.	UNICEF, 2013
Child disability level	Categorical measure indicating the index child’s level of difficulty with 11 functional domains as reported by the caregiver: vision, hearing, mobility, self-care, communication, learning, remembering, focusing attention, coping with change, controlling behavior, and relationships and emotions (making friends)	• 22 items assessing the index’s child’s level of difficulty using a Likert scale rating (no difficulty, some difficulty, a lot of difficulty, cannot do at all) • Disability level is coded based on any functional difficulty, with children having no disability, mild disability (some difficulty), or moderate and/or severe disability (a lot of difficulty and/or cannot do at all)	• Does (name) have difficulty seeing? • Does (name) have difficulty hearing sounds like peoples’ voices or music? • Does (say name) have difficulty with self-care such as feeding or dressing him/herself?	Washington Group and UNICEF, 2016
Conflict-related traumatic experiences	Continuous measure (range: 0–5) summing the number of affirmative responses to experiences of conflict-related acts	• 5 binary items (yes/no) • Any affirmative binary response (yes) was coded as experiencing that form of conflict-related act • Responses tallied to form a continuous summary measure	During any times of conflict, has someone in your family or someone close to you: • ever been seriously injured?	WHO and GWI, 2017
Disability attitudes	Continuous measure (range: 0–22) summing the number of statements with which a respondent agreed (2), partially agreed (1), or disagreed (0)	• 11-item Likert scale (agree, partially agree, disagree) • Higher score denotes more agreement with discriminatory attitudes toward people women and children disabilities	• It is natural for a man to beat his wife with a disability because she cannot be a good wife. • It is less important for a child with a disability to have an education than a child without a disability.	Developed for the study

Hypothesized correlates included conflict-related traumatic experiences and disability attitudes (Table 1). Conflict experiences is a measure adapted from the World Health Organization (WHO) Survey on Situation of Health and Life Experience for use on conflict settings (Global Women’s Institute & WHO, 2017). Disability attitudes was a measure developed for this study based on formative qualitative research conducted with men and women in eastern DRC on risks of violence in the household (Falb et al., 2022), the items for which can be found in Table 2. Caregiver demographic measures included continuous measures of age in years and number of times displaced in their lifetime, as well as categorical measures of education level and disability level (Washington Group on Disability Statistics 2016). Child demographic measures were reported by adult caregivers and similarly included continuous measures of age in years and categorical measures of gender, education level, and disability level (WG and UNICEF, 2016). Child disability level (Table 1) was determined using the caregiver’s reporting of the index child’s level of difficulty with 11 core functions (Table 3). Children were coded as having no disability, mild disability (some difficulty), or moderate and/or severe disability (a lot of difficulty and/or cannot do at all). While the UNICEF and Washington Group child disability module was designed for use with women as the primary caregiver, for this study it was applied in both the women’s and men’s questionnaire to assess each parent’s perception of their child’s difficulty functioning.

**Table 2:** Parent and child demographics, disability attitudes and perpetration of violent discipline against children, as reported by women (N = 198) and men (N = 196)

	Women (N = 198)		Men (N = 196)	
	Overall sample Col % (N) Mean (SD)	Perpetration of physical and/or psychological violent discipline Row % (N) Mean (SD)	Overall sample Col % (N) Mean (SD)	Perpetration of physical and/or psychological violent discipline Row % (N) Mean (SD)
Overall	-	83.84 (166)	-	74.49 (146)
**Parental Demographics**				
Parent age Mean (SD) (range: 19–59)	32.0 (8.7)	31.7 (8.5)	36.9 (8.7)	36.0 (8.5)
Parent education level None Primary Secondary and above	42.9 (85) 41.9 (83) 15.15 (30)	88.2 (75) 79.5 (66) 83.3 (25)	12.2 (24) 44.4 (87) 43.4 (85)	87.5 (21) 79.3 (69) 65.9 (56)
Parent disability level None Mild Moderate/Severe	21.2 (42) 60.1 (119) 18.7 (37)	83.3 (35) 79.8 (95) 97.3 (36)	39.3 (77) 42.4 (83) 18.4 (36)	70.1 (54) 74.7 (62) 83.3 (30)
Number of times displaced in lifetime Mean (SD) (range: 0–10)	3.18 (1.5)	3.2 (1.5)	3.0 (1.1)	3.1 (1.2)
Conflict-Related Traumatic Events Mean (SD) (range: 0–5)	3.6 (1.5)	3.8 (1.5)	3.3 (1.5)	3.3 (1.5)
**Child Demographics**				
Child age Mean (SD) (Range: 6–12)	10.01 (2.38)	9.95 (2.41)	9.82 (2.31)	9.91 (2.26)
Child gender Boys Girls	45.96 (91) 54.04 (107)	78.01 (71) 88.79 (95)	44.39 (87) 55.61 (109)	77.01 (67) 72.49 (79)
Child disability level^i^ None Mild Moderate/Severe	27.78 (55) 47.98 (95) 24.24 (48)	76.36 (42) 84.21 (80) 91.67 (44)	45.41 (89) 44.90 (88) 9.69 (19)	67.42 (60) 81.82 (72) 73.68 (14)
**Girl Children**				
Girls disability level None Mild Moderate/Severe	28.04 (30) 46.73 (50) 25.23 (27)	76.67 (23) 94.00 (47) 92.59 (25)	44.04 (48) 44.04 (48) 11.92 (13)	64.58 (31) 79.17 (38) 76.92 (10)
**Boy Children**				
Boys disability level None Mild Moderate/Severe	27.47 (25) 49.45 (45) 23.08 (21)	76.00 (19) 73.33 (33) 90.48 (19)	47.13 (41) 45.98 (40) 6.90 (6)	70.73 (29) 85.00 (34) 66.67 (4)
**Disability Attitudes**				
Disability attitudes Mean (SD) (Range: 0–22)	10.76 (4.50)	11.03 (4.27)	6.30 (4.22)	6.72 (4.23)
**Partially agree or agree with disability attitudes items**				
1. Women with disabilities cannot be victims of domestic violence because they don’t engage in relationships in the first place.	64.65 (128)	87.50 (112)	40.31 (70)	78.48 (62)
2. When a woman with a disability has been forced to have sex against her will, she should seek help immediately.	78.28 (155)	85.81 (133)	82.65 (162)	72.84 (118)
3. It is natural for a man to beat his wife with a disability because she cannot be a good wife.	27.27 (54)	85.19 (46)	17.35 (34)	85.29 (29)
4. A woman with an intellectual disability who says she experienced violence should be believed.	57.07 (113)	84.96 (96)	69.90 (137)	73.72 (101)
5. A woman with a disability who experiences violence should not report it to authorities because it will bring shame on her family.	64.65 (129)	85.94 (110)	45.41 (89)	78.65 (70)
6. Physical and sexual violence against women with disabilities can be prevented.	90.40 (179)	83.80 (150)	92.96 (182)	75.27 (137)
7. A child with disabilities should stay at home because if they play with other kids or participate in activities outside the home, they may be hurt.	84.34 (167)	85.03 (142)	51.53 (101)	74.26 (75)
8. A child born with a disability brings shame upon their family.	43.94 (87)	91.95 (80)	13.27 (26)	96.15 (25)
9. Children with disabilities who say they experience violence should not be believed.	55.56 (110)	85.45 (94)	21.43 (42)	90.48 (38)
10. A children with disabilities should only go to separate, more specialized services designed for children with disabilities.	84.34 (167)	86.83 (145)	57.65 (113)	80.53 (91)
11. It is less important for a child with a disability to have an education than a child without a disability.	56.06 (111)	90.09 (100)	20.92 (41)	82.93 (34)

^i^ Child disability level is defined as none, mild (some difficulty), moderate (a lot of difficulty) and/or severe (cannot do at all) with core functions in the Washington Group/UNICEF module on Child Functioning. Core functions include seeing, hearing, mobility, self-care, communication, learning, remembering, focusing attention, coping with change, controlling behavior, and making friends

**Table 3:** Children with moderate and/or severe difficulty in functional domains by child gender, as reported by women (N = 198) and men (N = 196)

	Women			Men		
	Overall (N = 198)	Boy Children (N = 91)	Girl Children (N = 107)	Overall (N = 196)	Boy Children (N = 87)	Girl Children (N = 109)
**Moderate and/or Severe Functional Difficulty among Children** ^**ii**^						
Seeing	2.54 (7)	2.20 (2)	4.67 (5)	0.51 (1)	1.15 (1)	0.00 (0)
Hearing	0.51 (1)	0.00 (0)	0.93 (1)	0.51 (1)	0.00 (0)	0.92 (1)
Mobility	2.02 (4)	2.20 (2)	1.87 (2)	4.08 (8)	2.30 (2)	5.50 (6)
Self-care	4.04 (8)	4.40 (4)	3.74 (4)	1.53 (3)	1.15 (1)	1.83 (2)
Communication	0.51 (1)	1.10 (1)	0.00 (0)	1.02 (2)	1.15 (1)	0.92 (1)
Learning	2.53 (5)	4.40 (4)	0.93 (1)	1.53 (3)	1.15 (1)	1.83 (2)
Remembering	5.05 (10)	3.33 (3)	6.54 (7)	2.04 (4)	1.15 (1)	2.75 (3)
Focusing attention	2.53 (5)	4.40 (4)	0.93 (1)	1.02 (2)	0.00 (0)	1.83 (2)
Coping with change	6.57 (13)	8.79 (8)	4.67 (5)	2.04 (4)	0.00 (0)	3.67 (4)
Controlling behavior	8.59 (17)	9.89 (9)	7.48 (8)	1.02 (2)	0.00 (0)	1.83 (2)
Making friends	2.53 (5)	2.20 (2)	2.80 (3)	1.02 (2)	1.15 (1)	0.92 (1)
**Moderate and/or Severe Psychosocial Difficulty among Children**						
Anxiety	37.88 (75)	40.66 (37)	35.51 (38)	26.02 (51)	29.89 (26)	22.94 (25)
Depression	39.39 (78)	38.46 (35)	40.19 (43)	24.49 (48)	26.44 (23)	22.94 (25)

^ii^ To assess the child’s functional difficulty, each parent was asked whether the child had a lot of difficulty with 11 domains. Those with moderate and/or severe functional difficulty are those who have a lot of difficulty or cannot do them at all with any of the domains. Psychosocial measures were not included in the calculation of functional difficulty

### Analysis

The paper first examines the socio-demographic characteristics of the participants overall, then by perpetration of violent discipline. Demographic data and overall frequencies or means were assessed for women and men separately. Hypothesized correlates were then assessed through unadjusted models in gender-stratified analyses. Those covariates that were significantly associated with perpetration of violent discipline at p < .05 for either men or women were retained in the adjusted models. Logistic generalized estimating equations were used to assess the adjusted association between correlates and perpetration of violent discipline, accounting for clustering at the site level and adjusted for other correlate variables and caregiver demographics. The variables used in these analyses had no missing data.

## Results

### Descriptive Statistics

Descriptive statistics are stratified by gender of the adult respondent and the outcome of violent discipline in Table 2. Reporting of child gender and disability level for the index child differed between men and women within couples, with men’s reporting of their child having a moderate and/or severe disability lower overall (9.7% of men compared to 24.2% of women). The stratification of type of child disability by functional domain can be found in Table 3, stratified by gender of the adult respondent and overall reporting of functional difficulty and gender of the child. In supplemental analyses, types of disability had little overlap (reports of multiple types of disability), with exception of cognitive and behavioral functional difficulties. Anxiety and depression overlapped with multiple types of child disability, with caregivers reporting that their child had anxiety and/or depression as well as another type of disability.

### Child Maltreatment Within the Home

In binary measures, perpetration of physical and/or psychological violent discipline was high overall, with approximately four out of five (83.8%) women and three out of four men (74.5%) perpetrating violent discipline in the past three months (Table 2). Respondents reported high poly-victimization of violence, with statistically significant overlap of physical and psychological violent discipline reported by both women (p < .0001) and men (p < .0001) for perpetration against both boy and girl children. Among women, more women with girl children (88.8%) reported perpetrating physical and/or psychological violent discipline compared to those with boy children (78.0%). Reported perpetration of violent discipline also varied by disability level of the child, with highest perpetration reported among women whose children had a moderate and/or severe disability (91.7%). In contrast, slightly fewer men reported perpetrating violent discipline against girl children (72.5%) compared to those with boy children (77.0%). Among men who reported their child having a disability, more men with a child with a mild disability (81.8%) reported perpetrating violent discipline compared to those with a child with a moderate and/or severe disability (73.7%).

### Women’s Perpetration of Violent Discipline in the Past Three Months

#### Unadjusted associations between past-three-month violent discipline, child gender, child disability, and other child and parent demographics

In unadjusted logistic regressions accounting for clustering at the site level (Table 4), child disability level was strongly associated with perpetration of physical and/or psychological violent discipline, such that women with children with moderate or severe disability had 3.10 times the odds of perpetrating violent discipline to those with no disability (95%CI: 1.48–6.51) and women with children with mild disability had 1.68 times the odds of perpetrating violent discipline to those with no disability (95%CI: 1.10–2.59). Other child demographic variables of gender or age were not significantly associated with perpetration of violent discipline. Women’s disability attitudes were also not significantly associated with reported perpetration of violent discipline in the past three months.

**Table 4:** Unadjusted associations of child gender, child disability and past-three-month perpetration of violent discipline against children, as reported by women (N = 198) and men (N = 196)^i^

	Women	Men
	Physical violent discipline and/or Psychological violent discipline OR (CI), p-value	Physical violent discipline and/or Psychological violent discipline OR (CI), p-value
**Child demographics**		
Child age Mean (SD) (Range: 6–12)	0.93 (0.85, 1.01), 0.7200	1.07 (0.98, 1.17), 0.1575
Child gender (Ref.=Boy) Girl	2.19 (0.80, 5.99), 0.1254	0.75 (0.45, 1.24), 0.2607
Child disability level (Ref.=none) Mild Moderate/Severe	1.68 (1.10, 2.59), 0.0173 3.10 (1.48, 6.51), 0.0028	2.00 (0.79, 5.08), 0.1445 1.24 (0.42, 3.67), 0.7026
**Parent demographics**		
Parent age	0.98 (0.94, 1.02), 0.3532	1.01 (0.94, 1.04), 0.7571
Parent education level (Ref.=None) Primary Secondary and above	0.49 (0.30, 1.14), 0.0962 0.69 (0.48, 0.995), 0.0472	0.56 (0.07, 4.25), 0.5765 0.29 (0.08, 1.08), 0.0652
Parent disability level (Ref.=None) Mild Moderate/Severe	0.79 (0.41, 1.54), 0.4921 7.46 (2.11, 26.34), 0.0018	1.13 (0.45, 2.80), 0.7954 1.98 (1.61, 2.43), <0.0001
**Parent conflict experiences**		
Conflict-Related Traumatic Events	1.42 (1.19, 1.69), 0.0001	1.06 (0.99, 1.13), 0.0813
Number of times displaced in lifetime	1.14 (0.99, 1.32), 0.0664	1.10 (0.80, 1.52), 0.5417
**Parent disability attitudes**		
Disability attitudes Mean (SD) (Range: 0–22)	1.07 (0.99, 1.17), 0.0990	1.10 (1.04, 1.16), 0.0006

^i^ Adjusted for clustering at the site level

^ii^ Psychosocial difficulty includes those children with reported moderate and/or severe anxiety or depression

Parental demographics had mixed significance of association with child maltreatment. Parent moderate or severe disability level was significantly associated with perpetration of violent discipline (OR = 7.46; 95%CI: 2.11–26.34), but reporting a mild disability was not significantly associated with child maltreatment. Women with secondary education or above reported significantly lower odds of perpetrating violent discipline in the past three months than those with no education (OR = 0.69; 95%CI:0.48–0.995), but the association with reporting primary education was not significant. Women’s own conflict experiences were significantly associated with violent discipline, with each additional increase in reporting a conflict-related traumatic experience associated with a 1.42 increase in odds of reporting perpetration of violent discipline in the past three months (95%CI: 1.19–1.69). Parent age and lifetime displacement had no significant association with perpetration of violent discipline and were thus removed from the final adjusted model.

#### Adjusted Associations Between Past-three-month Violent Discipline and the Interaction of Child Gender and Child Disability

**Fig. 1:**
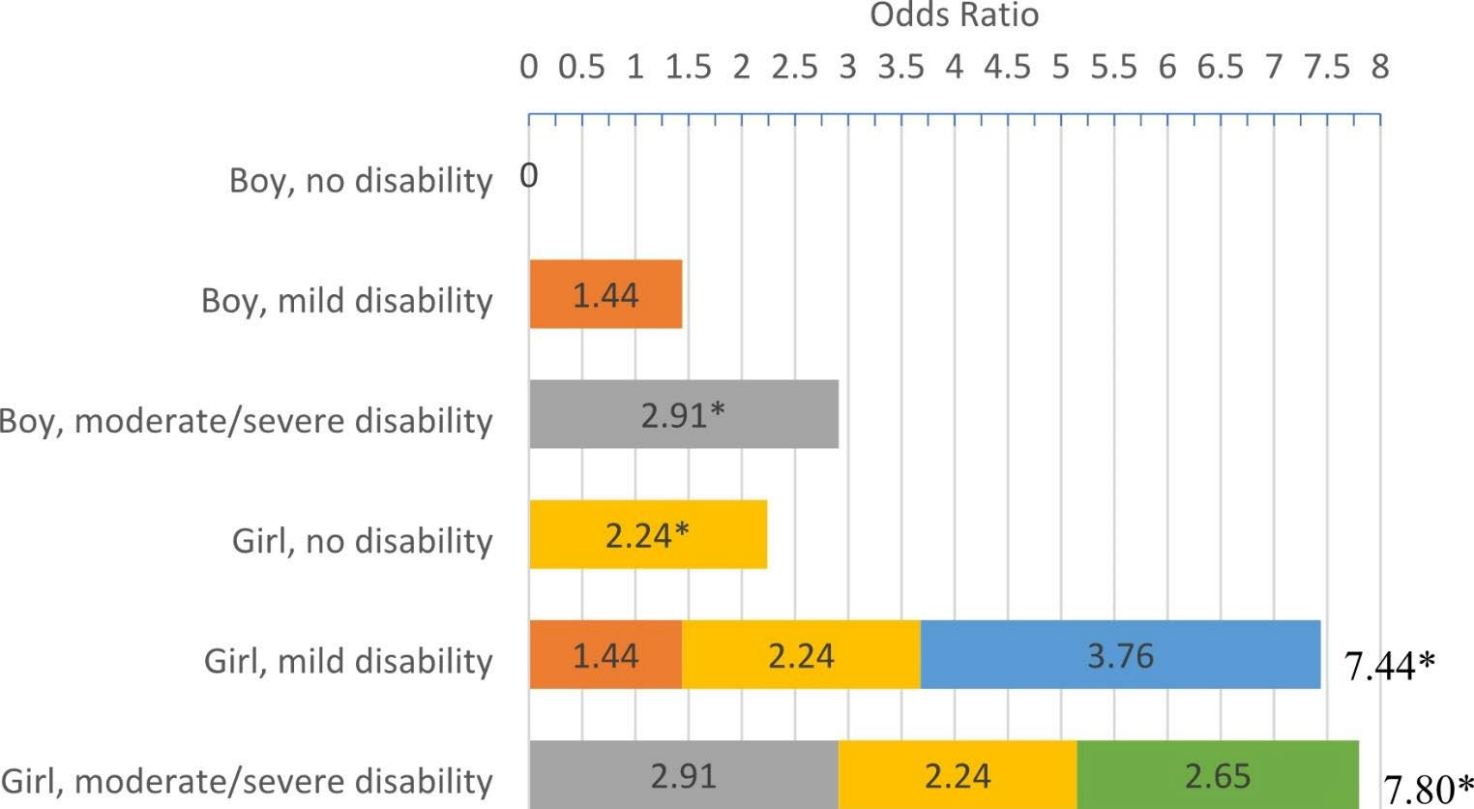
Increased odds of perpetration of violent discipline by the interaction of child gender and disability level (reference = boy, no disability) as reported by women who reported perpetrating violent discipline (N = 166). (*Notes statistical significance at p < .05 level)

After accounting for other statistically significant caregiver demographics, hypothesized correlates, and clustering at the site level, the adjusted model examined the interaction of child gender and disability level on the odds of perpetration of violent discipline (Table 5). Within this model, compared to the reference group of boy children with no disability, women reporting on girl children with a moderate or severe disability had the highest statistically significant increase in odds of perpetrating violent discipline: 7.80 increase in odds (Fig. 1). This finding was followed by women reporting on girl children with a mild disability, who had a statistically significant 7.44 increase in odds of perpetrating physical and/or violent discipline in the past three months. Women who reported their boy child having a moderate or severe disability had statistically significant 2.91 (95%CI: 1.14–7.33) increase in odds of perpetrating violent discipline in the past three months compared to those with boy children with no disability (Table 5; Fig. 1). Examining the simple effects of gender in the model, women reporting on girl children with no disability had a statistically significant 2.24 (95%CI: 1.11–4.51) increase in odds of perpetrating violent discipline compared to those reporting on boy children with no disability. However, the simple effect of boy children with mild disability was no longer significantly associated with violent discipline perpetration in the adjusted model. The association of parent conflict-related traumatic events remained significant within the adjusted model (aOR = 1.53, 95%CI: 1.29–1.82). Women’s discriminatory disability attitudes remained non-significant in its association with perpetration of violent discipline in the past three months within the adjusted model.

**Table 5:** Adjusted associations of child gender, child disability and past-three-month perpetration of violent discipline against children, as reported by as reported by women (N = 198) and men (N = 196)^i^

	Women	Men
	Physical violent discipline and/or Psychological violent discipline aOR (CI), p-value^ii^	Physical violent discipline and/or Psychological violent discipline aOR (CI), p-value^iii^
**Child gender (Ref.=Boy)** Girl	2.24 (1.11, 4.51), 0.0238	0.92 (0.57, 1.46), 0.7079
**Child disability level (Ref.=none)** Mild Moderate/Severe	1.44 (0.81, 2.54), 0.2119 2.91 (1.14, 7.33), 0.0238	2.04 (0.75, 5.60), 0.1656 1.06 (0.25, 4.42), 0.9377
**Interaction of child gender and child disability level (Ref.=Boy, no disability)** Girl, Mild Girl, Moderate/Severe	3.76 (1.04, 12.67), 0.0441 2.65 (1.02, 6.92), 0.0464	1.96 (0.85, 4.50), 0.1146 1.38 (0.33, 5.69), 0.6593
Conflict-Related Traumatic Events	1.53 (1.29, 1.82), <0.001	1.08 (0.96, 1.22), 0.2092
Disability attitudes Mean (SD) (Range: 0–22)	1.10 (0.93, 1.29), 0.2553	1.07 (1.00, 1.15), 0.0421

^i^ Adjusted for statistically significant parent demographics and clustering at the site level

^ii^ Final N for women’s models: Perpetration of physical and/or psychological violent discipline (N = 166)

^iii^ Final N for men’s models: Perpetration of physical and/or psychological violent discipline (N = 146)

### Men’s Perpetration of Violent Discipline in the Past Three Months

#### Unadjusted Associations Between Past-three-month Violent Discipline, Child Gender, Child Disability, and Other Child and Parent Demographics

In unadjusted logistic regressions controlling for clustering at the site level, violent discipline was not significantly associated with hypothesized correlate variables of child gender, child disability, or child’s age and men’s reported perpetration of violent discipline in the past three months (Table 4). However, each higher acceptance of harmful disability attitudes was significantly associated with a 1.10 (95%CI: 1.04–1.16) increase in odds of perpetrating violent discipline.

Moderate and/or severe disability level among men was strongly associated with perpetration of violent discipline (OR = 1.98; 95%CI: 1.61–2.43), but reporting a mild disability had no significant association. In contrast to women, no other caregiver variables for men were significantly associated with their perpetration of violent discipline in the past three months.

#### Adjusted Associations Between Past-three-month Violent Discipline, Child Gender, Child Disability, and Other Child and Parent Demographics

The final adjusted model—accounting for statistically significant parent demographics and clustering at the site level—produced very different results for men as compared to women. The interaction of child gender and child disability were not significantly associated with perpetration of violent discipline in the past three months, nor were the simple effects of child gender and child disability. Disability attitudes remained significantly associated with men’s violent discipline perpetration, with every 1-unit increase in discriminatory disability attitudes among men increasing their odds of perpetration of violent discipline 1.07 times (aOR: 1.07; 95%CI: 1.00-1.15). Men’s conflict experiences were not significantly associated with past-three-month perpetration of violent discipline in the adjusted models.

## Discussion

The results of the study reveal the levels of violent discipline against children within conflict-affected households in North Kivu, DRC. Overall, approximately three out of four families reported any violent discipline by both partners within the past three months, with higher perpetration reported among women. Women reporting higher levels of violent discipline perpetration is supported by evidence which highlights that women may perpetrate more violence against children as they spend more time parenting and may have higher levels of parenting-related stress (May-Chahal & Cawson, 2005; Niu et al., 2018). Women’s perpetration of violence as a disciplinary practice could also relate to their experiences of violence in childhood (Slegh et al., 2014), in addition to being a potential protective measure to try to reduce IPV used against them by their partner as a punishment for the child’s behavior (Fulu et al., 2017). Evidence linking IPV to perpetration of violent discipline could further explain why women who reported having a moderate or severe disability had higher odds of perpetrating violence. Recent studies from eastern DRC and other conflict settings have demonstrated that women and girls with disabilities are at higher risk of experiencing IPV (Scolese et al. 2020; van der Heijden & Dunkle, 2017).

The study also revealed women’s amplified use of violent discipline practices against girl children with disabilities. Compared to those with boys with no disability, women were more likely to use physical and/or psychological violent discipline against girls and children with disabilities, with a multiplied effect for girl children with disabilities. While some studies have found that boys experience higher levels of physical violence in the home than girls (Maternowska et al., 2016), these findings are consistent with other studies that have found that girls experience high levels of abuse from caregivers and other family members in humanitarian settings (Stark et al., 2020), and girls with disabilities are more likely to experience physical and sexual violence than boys with disabilities (Devries et al., 2015). This finding differs slightly from a qualitative study conducted in DRC which identified the perception that children with disabilities were less likely to face maltreatment due to protective norms such as compassion for persons with disabilities (Falb et al., 2022). However, the finding that women are more likely to perpetrate violence against girl children with disabilities could reflect the effect of stigma and potential stressors of parenting a child with a disability in a resource-constrained context layered with dissonant attitudes toward gender. Women’s gender equitable attitudes have been found to be associated with lower risk of violence for adolescent girls, more so than attitudes and beliefs relating to parenting and harsh discipline (Falb et al., 2017). Therefore, women’s own negative gender attitudes could influence their perpetration of violent discipline against children.

Notably, men’s perpetration of violent discipline was not significantly associated with any child demographic factors including child gender and disability level. This finding, and the discrepancy between women and men’s reporting of disability level for the index child, could also relate to men’s reduced time with their children relative to women, nullifying hypotheses around child-level risk factors of violence. In a qualitative study among conflict- and drought-affected communities in Kenya, men were absent in more than half of families, which women commonly explained was due to stigma around having a disabled child (Zuurmond et al. 2016). There may also be constructs missing from the model, such as household income and stress and anger or emotional regulation, to which men may be more susceptible than women (Falb et al., 2022). Other evidence has found that among conflict-affected populations, men’s own mental health is also associated with higher levels of violent discipline (Saile et al., 2014).

Given the significantly higher odds of perpetration against girl children with disabilities by women caregivers, these findings highlight the need for research and programs on violent discipline to apply an intersectional lens and actively seek to understand and address the multiple and compounding risk factors faced by both children and their caregivers that influence violence within the home. The influence of gendered perpetration of violence against children illuminates the need for family-based interventions to not only build parenting skills, but also to specifically address underlying inequitable norms around gender and disability (Falb et al., 2022). Such an approach could reduce harmful gender and disability attitudes that result in higher violence against girls and children with disabilities while simultaneously emphasizing the use of non-violent discipline. Literature demonstrates that addressing gender inequitable attitudes can reduce GBV at the hands of intimate partners, caregivers, and other perpetrators (Bacchus et al., 2017; Stark et al., 2020). A similar approach could be taken with addressing harmful disability attitudes to reduce violence against children with disabilities (Marshall & Barrett, 2018; van der Heijden & Dunkle, 2017).

Similarly, the majority of family-based programs involve only women as the primary caregiver, reenforcing harmful gender norms around the role of caregiving as being a woman’s responsibility. This is reflected in the findings from this study showing differences in perpetration of violence by women and men, likely related to women being the primary caregiver of children. These findings emphasize the need to engage men in interventions to reduce violence against children (Ashburn et al. 2017), as such an approach could serve to reduce perpetration of violent discipline by both women and men while also influencing norms around caregiving as a shared responsibility between partners within the home.

These analyses have several limitations which should be considered in their interpretation. First, the study is lacking the voices of children for whom the intervention aims to reduce violence and improve outcomes of wellbeing. Children were not engaged in the study due to ethical, logistical, and budgetary considerations. Other literature examining both child- and caregiver-reported data have found meaningful differences in prevalence of harsh discipline, and future research on families should include child voices where safe and ethical to do so. Second, study outcomes, including child demographics, were self-reported by adult women and men, leaving the potential for underreporting of violence perpetration due to desirability bias. Third, this paper aims to specifically test the hypothesis of the association between child demographics and perpetration of violent discipline, and for that reason the models did not include higher level factors that may also influence violence perpetration, such as household stress (Falb et al., 2022). Finally, the analyses draw from the baseline data of an ongoing trial, in which participants had to agree to partake. This procedure may reflect a bias among households with both partners in a couple who were motivated to improve their family functioning, thereby limiting generalizability of the findings to other households within the community and across other conflict settings.

## Data Availability

Available upon request from the lead author.

## Code Availability

Data were analyzed using SAS 9.4 for Windows. Coding procedures for key measures are detailed in the manuscript.
